# A new era in solar system astronomy with JWST

**DOI:** 10.1038/s41467-023-43313-z

**Published:** 2023-11-17

**Authors:** G. L. Villanueva, S. N. Milam

**Affiliations:** NASA Goddard Space Flight Center, Greenbelt, MD USA

**Keywords:** Astrobiology, Astronomical instrumentation, Space physics

## Abstract

The exploration of our solar system is being radically changed since the beginning of operations of the James Webb Space Telescope (JWST) in mid 2022. JWST’s extraordinary sensitivity and instrumentation allow for sensitive searches for the building blocks of life and to test for habitability, also enabling new discoveries on small bodies to giant planets across our solar system and beyond.

## Solar system exploration

An important theme in the exploration of our solar system has been the search for life and habitability beyond our planet, and the associated investigation of how internal and external processes impact the formation and evolution of planetary bodies. The search relies on the accurate characterization of several fundamental chemical markers (e.g., water, carbon dioxide, ammonia, organics) that serve as the building blocks of life. Many of these species have strong infrared signatures observable with JWST, while geological and evolutionary processes can leave distinctive observable markers across the surfaces and atmospheres of our solar system. We then ask, where do water and organics in our planet originate from? If the building blocks of life were delivered to Earth by small bodies, are those molecular components present in other objects, or were they also delivered across the solar system? Deciphering these questions requires a systematic and detailed investigation of objects in our solar system, including the highly evolved terrestrial planets (e.g., Mars), the most primordial objects tracing back to the proto-solar nebula (e.g., comets, trans-Neptunian objects), and the gas (Jupiter and Saturn) and icy (Uranus and Neptune) giants, which are the main repositories of volatile species in our solar system. When searching for actual signatures for life and habitability, the fascinating ocean worlds (e.g., Jupiter’s moon Europa and Saturn’s moon Enceladus), the organic-rich moon Titan, and Mars, all share potentially habitable conditions.

A powerful way to address these questions is via sensitive remote sensing employing space-based and ground-based observatories. For instance, optical and ultraviolet observations with the Hubble Space Telescope (HST) have enabled the remote exploration of these objects with high spatial resolution and sensitivity^1^, while primarily focusing on imaging and atomic signatures. Previous infrared space observatories (e.g., Spitzer, Herschel) pierced into wavelengths inaccessible to ground-based observatories and demonstrated the high value of planetary astronomy at longer wavelengths^2^. The study of astrobiologically relevant species with ground-based observatories is often quite challenging, namely due to the strong presence of these key species in our atmosphere, and therefore restricting detections remotely. Otherwise, impressive ground-based adaptive optics capabilities with ground-based observatories (e.g., Keck, Very Large Telescope) have allowed to explore faint and distant bodies with unprecedented spatial resolutions in the optical and infrared^3^, while advanced high-resolution spectrometers and powerful interferometric radio observatories (e.g., Atacama Large Millimeter Array) have allowed to sensitively probe for organic and volatile species across the solar system in specific spectral windows^4^.

## A new era in planetary research

**Fig. 1:**
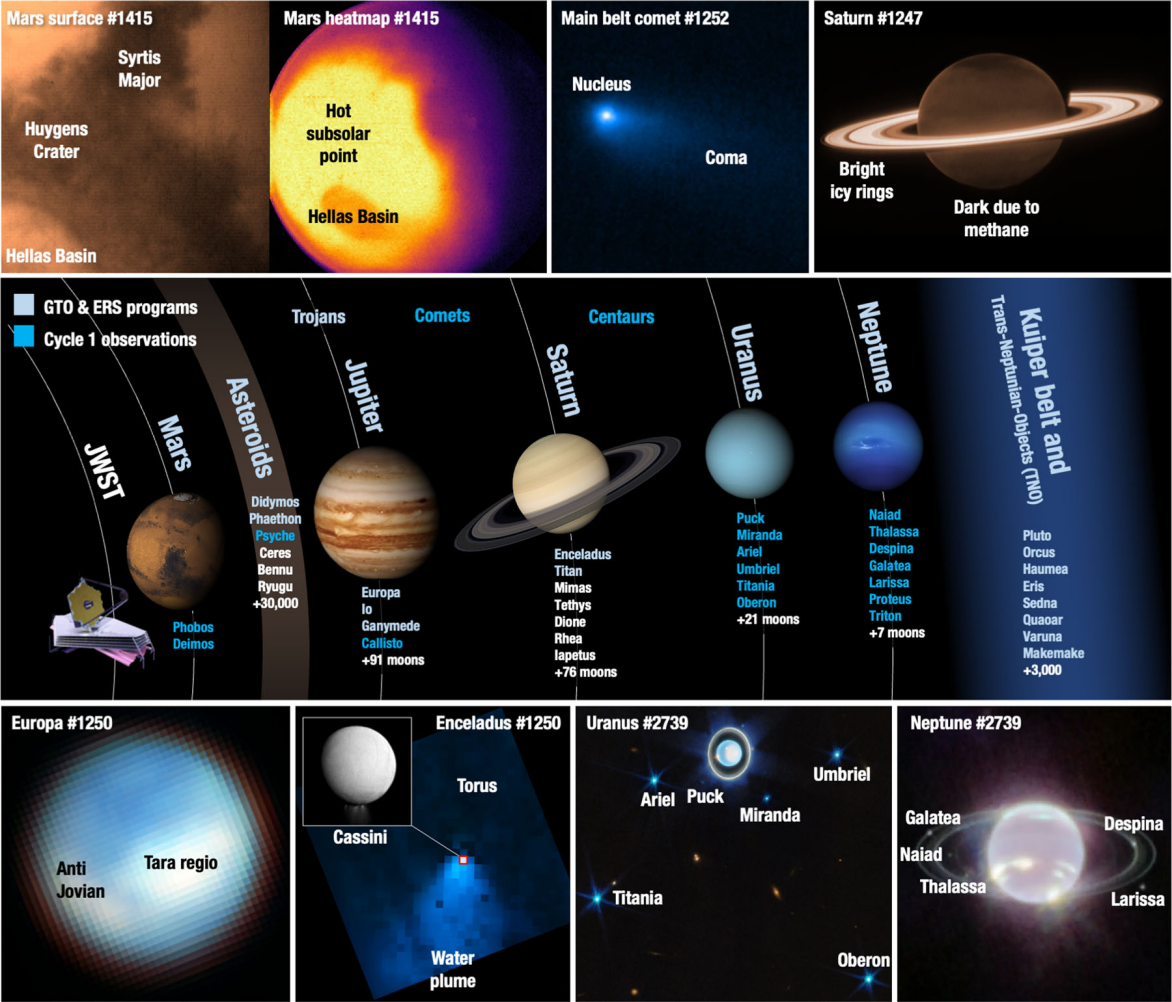
Collage of several images of solar system objects as observed with JWST. In the first year of operations, JWST has been used to study a wide range of solar system objects (see Table 1), setting the foundations for many future unique investigations. Program IDs are listed for each publicly released image. The observations sample the faintest, the smallest, the brightest, the biggest, and the fastest moving (e.g., near-Earth asteroids) bodies in our solar system, and employ a wide variety of instrument modes, covering a wide range of wavelengths at extraordinary sensitivities. Such a large dynamic range of available capabilities is essential for solar system research, and ultimately provide unique information regarding the origin, evolution and state of our planetary system.

Yet in the summer of 2022, with the beginning of operations for JWST, solar system astronomy was radically changed. A cryogenic space observatory with state-of-the-art imagining and spectroscopic instrumentation across the near to mid-infrared spectral range permits to explore planetary objects with sensitivities many orders of magnitude better than previous generation facilities^5–7^. Uniquely at infrared wavelengths, the fine spatial resolution delivered with a large 6.5 meters diffraction limited aperture, enables extraordinary mapping and characterization of planetary surfaces and atmospheres. This capability allows for careful searches of regions of active volatile release, as well as to obtain detailed maps of the dynamical processes acting on the planets Mars, Jupiter, Saturn^8^, Uranus and Neptune (see Fig. 1 and Table 1). Such mapping capabilities are particularly critical when studying many of ocean worlds^9–11^, comets^12^, Jovian moons^13,14^, embedded moonlets, binary objects, and tightly packed ring systems. JWST has also demonstrated the ability to track a fast dynamical Double Asteroid Redirection Test (DART) impact event (Table 1), with a tracking rate over three times faster than for the prelaunch capabilities offered. This permits for detailed studies of small bodies in the inner solar system, such as near-Earth asteroids or active comets, that are still in the JWST field of regard. Most of these first observations took place under the Guaranteed Time Observations (GTO) and Early Release Science (ERS) programs, or as part of the general community annual cycle 1 observations. Many other novel and continued observations will take place in the following cycles (e.g., cycle 2: https://tinyurl.com/5benhajh), which rely on an annual open call for proposals.

**Table 1:** Selected list of recent JWST press releases and articles reporting on solar system objects

Team (program ID)^a^	Targets	Articles and press releases^b^
Thomas et al. (GTO 1245) https://tinyurl.com/yc69uc6n	Near-Earth-Objects and DART mission support	Press https://tinyurl.com/2mex35b5
Fletcher et al. (GTO 1247) https://tinyurl.com/4sv3ax4h	Saturn	Published article (8) Press https://tinyurl.com/2p96ce2y
Villanueva et al. (GTO 1250) https://tinyurl.com/2uem9967	Enceladus and Europa	Published articles (9–11) Press https://tinyurl.com/msps6zax Press https://tinyurl.com/5wr6mxv5
Nixon et al. (GTO 1251) https://tinyurl.com/mk295zr7	Titan	Press https://tinyurl.com/292mt7tj
Kelley et al. (GTO 1252) https://tinyurl.com/y66kp569	Comets	Published article (12) Press https://tinyurl.com/3jdprrnd
Santos-Sanz et al. (GTO 1271) https://tinyurl.com/mw8asxd2 Hines et al. (GTO 1272) https://tinyurl.com/mwn7n4zd	Rings, KBOS, and TNOS	Press https://tinyurl.com/y3dpjftv
de Pater, Fouchet et al. (ERS 1373) https://tinyurl.com/38h6ztz6	Jupiter and moons	Published articles (13,14) Press https://tinyurl.com/22ruheut Press https://tinyurl.com/yvw9kc7f
Villanueva et al. (GTO 1415) https://tinyurl.com/nhermd3v	Mars	Press https://tinyurl.com/yck56xxh
Pontoppidan et al. (Director’s Discretionary Time [DDT] 2739)	Neptune, Uranus, and other targets	Press https://tinyurl.com/y2u5p6uv

^a^The links under the “team” column provide full information on the allocated programs and the associated observation methods and strategies.

^b^The publicly available images and press-releases can be accessed via the “press” links, with several of the released images shown in Fig. 1.

**Fig. 2:**
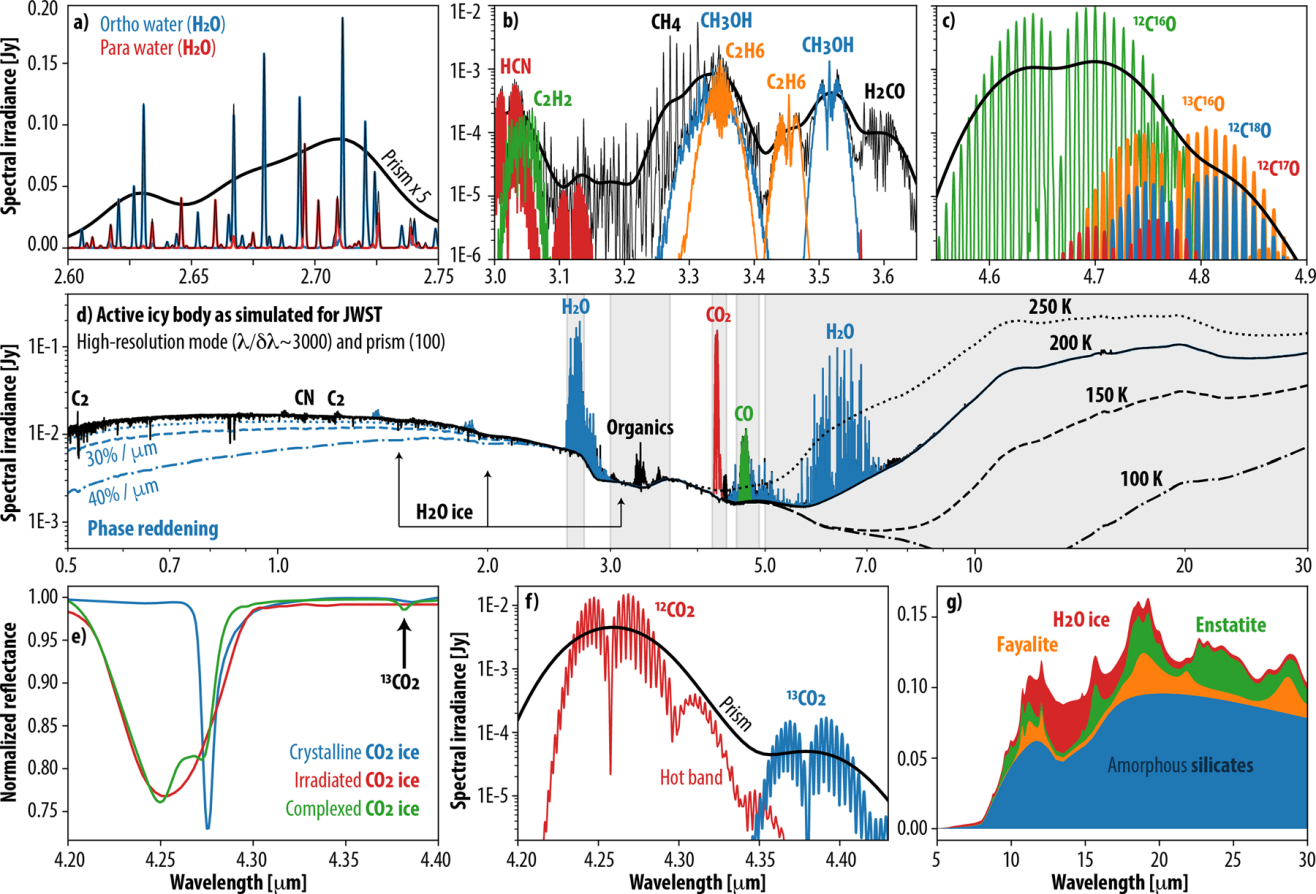
Synthetic spectra of a hypothetical active icy body as measured with JWST. This telescope provides unparalleled access to the infrared (0.6 to 28.3 μm) spectrum of solar system objects, allowing to sensitively probe many pre-biotic molecular tracers, and to study key geological/evolutionary processes. The high-resolution mode of JWST/NIRSpec resolves the strong infrared ortho/para lines of H_2_O **a**, a plethora of organic species **b**, and several isotopic ro-vibrational lines of CO **c**, and of CO_2_ **f**. The sensitive low-resolution (R ~ 100) prism mode is shown with a thick black trace on **a**, **b**, **c**, **f**. Ices **d**, **e**, minerals **g**, temperature, and phase reddening **d** can be sensitively probed thanks to the wide spectral coverage of the instruments onboard JWST. The shown synthetic spectra have been generated using the Planetary Spectrum Generator (PSG, psg.gsfc.nasa.gov)^22^ for a small (7 km) active (10^28^ molecules/s) icy body at 1 AU assuming typical cometary abundances.

The revolutionary aspect of this new observatory is perhaps best revealed by the exceptional spectroscopic capabilities offered by JWST. Occasionally referred to as a molecular machine, JWST is uniquely primed for searching and detecting a plethora of gases, ices, and mineral features, thanks to the broad wavelength coverage (0.6 to 28.3 μm) and range of spectral capabilities from low to high resolution. A particularly notable capability is the combination of high-resolution spectra at each pixel obtained across 2-dimensional images with the IFUs (Integral Field Units). As shown in Fig. 2 for a hypothetical active icy object, many markers associated with prebiotic chemistry and evolutionary processes have strong signatures in this spectral range, which can be nicely separated and probed by the JWST instruments^5^: Near InfraRed Spectrograph (NIRSpec), Mid-Infrared Instrument (MIRI), Near Infrared Imager and Slitless Spectrograph (NIRISS), and Near Infrared Camera (NIRCam). For instance, JWST can sensitively probe several water (H_2_O) fundamental bands, and also probe the ortho/para ratio in water emission (Fig. 2a), a metric associated with the nuclear spin states of the hydrogen atoms of water and postulated as a diagnostic tracer of the formation temperature of primordial icy bodies^15^. Many strong water ice features are also present at 1.5, 2, 3, 6, and 13 μm, which dominate the spectra of many icy bodies (e.g., Europa, Enceladus), and help decipher the ice structure (e.g., crystalline, amorphous, irradiated)^16^, evolution and storage within a given body. In crystalline ice, the molecules are arranged in a highly regular pattern forming lattices (e.g., typical water ice on Earth), while in amorphous ice the molecules are aggregated irregularly. The latter is the most common form in the primordial interstellar medium and across the universe^17^.

## Searching for current activity and testing for evolution

Geological or biological activity, and evolutionary or formation processes over billions of years leave specific signatures on the materials they interact with, which can be tested by analyzing the chemical and mineralogical composition of planetary bodies. The busy C-H hydrocarbon region at 3.3 μm is sensitively resolved with the NIRSpec high-resolution mode enabling the distinction of many bands of various organic compounds, like methanol (CH_3_OH), ethane (C_2_H_6_), methane (CH_4_), formaldehyde (H_2_CO), acetylene (C_2_H_2_), and hydrogen cyanide (HCN) – A capability rarely available from space. Volcanic and thermal processes tend to produce a higher abundance of complex hydrocarbons relative to methane than biology, while the abundance ratio of several species can assist in exploring active cryovolcanism and its products. The mineralogy and ice composition of small bodies can be comprehensively studied at longer wavelengths with MIRI (Fig. 2g), where one can distinguish and identify between several solid-state features and sensitively test for temperatures and thermal properties (e.g., inertia/permittivity)^18^. At shorter wavelengths, JWST can effectively quantify the phase reddening effect, a phenomenon attributed to small-scale surface roughness on larger bodies, space weathering, and multiple scattering properties of dusty environments^19^.

A key spectral window now accessible with JWST is the region near 4.3 μm, where several gas and ice features of carbon dioxide (CO_2_) and its isotopologues are resolved (Fig. 2e, f). This wavelength region cannot be observed with ground-based facilities, and is only poorly resolved on some solar system objects by in-situ missions (e.g., Galileo, Cassini, Deep Impact).

On Earth, atmospheric CO_2_ is the main carbon source for biology and a dominant product of it, with the CO_2_ concentration in the pre-industrial era mostly set by life and geological processes. CO_2_ is also one of the main forms in which carbon is stored across the solar system (the other being CH_4_), with the CO_2_, H_2_O, CO, CH_4_ abundance ratios being highly diagnostic to the formation and evolution of primordial bodies because these volatiles have notably different sublimation/condensation temperatures. JWST can not only detect the CO_2_ gas and ice features, but it can also resolve their band shapes and structure. This is critical when exploring the state and conditions in which carbon is stored in these bodies and allowing us to disentangle key properties regarding the evolution, conversion, and stability of these volatile ices. For instance, irradiated CO_2_ amorphous ice looks completely different than the crystalline form^20^, and even more distinct when CO_2_ ice is stored in a complex organic matrix displaying a double peak shape (Fig. 2e)^21^. By mapping such signatures on icy moons and bodies across the solar system, one can potentially test the age of the exposed ices and search for cryogenic volcanism and regions of active volatile release. In addition, the capability of JWST to probe isotopic ratios in CO_2_ and CO (Fig. 2c/f) across the solar system would allow us to better understand the significance of these ratios as proxies to biological/geological/evolutionary processes.

## A revolutionary capability for the exploration of the outer solar system and beyond

The data collected in the last months have already demonstrated how ground-breaking JWST has been for solar system research (see Fig. 1 and Table 1), and many new investigations are now being planned for future cycles. Importantly, the pioneering science provided by JWST on astrobiology and evolutionary processes will not only be restricted to solar system research, but these findings will have strong implications on comparative planetology and the search for life and habitability beyond our system and in extrasolar planets. As we learn more about the diversity of molecular signatures and their connection to active processes in our solar system, we will be able to better correlate these to the data collected on exoplanets with JWST and future observatories. This together with the development of new powerful missions to probe the outer confines of our solar system and beyond, JWST will play a significant role in guiding the instrumentation and capabilities needed to effectively explore these distant worlds.

## Data Availability

All the data presented and described in this manuscript are available at the Mikulski Archive for Space Telescopes tool (MAST, mast.stsci.edu).
